# Effect of Underwater Insertion on Intracochlear Pressure

**DOI:** 10.3389/fsurg.2020.546779

**Published:** 2020-11-30

**Authors:** Conrad Riemann, Holger Sudhoff, Ingo Todt

**Affiliations:** Department of Otolaryngology, Head and Neck Surgery, Bielefeld University, Campus Mitte, Klinikum Bielefeld, Bielefeld, Germany

**Keywords:** intracochlear pressure, cochlear implant, cochlea model, hearing preservation, electrode insertion

## Abstract

**Background:** The importance of intracochlear pressure during cochlear electrode insertion for the preservation of residual hearing has been widely discussed. Various aspects of pre-insertional, intra-insertional, and post-insertional relevant conditions affect intracochlear pressure. The fluid situation at the round window during electrode insertion has been shown to be an influential factor.

**Aims/Objectives:** The aim of the study was to compare various insertion techniques in terms of the fluid situation at the round window.

**Material and Methods:** We performed insertion of cochlear implant electrodes in a curled artificial cochlear model. We placed and fixed the pressure sensor at the tip of the cochlea. In parallel to the insertions, we evaluated the maximum amplitude of intracochlear pressure under four different fluid conditions at the round window: (1) hyaluronic acid; (2) moisturized electrode, dry middle ear; (3) middle ear filled with fluid (underwater); and (4) moisturized electrode, wet middle ear, indirectly inserted.

**Results:** We observed that the insertional intracochlear pressure is dependent on the fluid situation in front of the round window. The lowest amplitude changes were observed for the moisturized electrode indirectly inserted in a wet middle ear (0.13 mmHg ± 0.07), and the highest values were observed for insertion through hyaluronic acid in front of the round window (0.64 mmHg ± 0.31).

**Conclusions:** The fluid state in front of the round window influences the intracochlear pressure value during cochlear implant electrode insertion in our model. Indirect insertion of a moisturized electrode through a wet middle ear experimentally generated the lowest pressure values. Hyaluronic acid in front of the round window leads to high intracochlear pressure in our non-validated artificial model.

## Introduction

The aim of modern cochlear implantation is intracochlear structural and functional preservation. The development of atraumatic electrodes decreased structural damage significantly. The performed surgical technique is assumed to highly influence functional preservation, which can vary due to inter-surgeon variability.

Electrode design, insertional force, tip size, intracochlear size, the insertion angle, and protective agents (e.g., steroids) are suggested contributing factors to the preservation of residual hearing (1, 2). Although the extended round window and cochleostomy approach have been shown to allow hearing preservation, most clinics use a round window approach related to a higher risk of labyrinthine damage by the two other approaches (3). Analyzing experimentally the various surgical steps during the procedure allows a classification of pressure-affecting operations. Factors before the insertion include the following: the size of the round window opening (4), the technique of round window opening (5), and the trans-fluid opening of the round window (6). Awareness of the importance of the round window's atraumatic opening has led to the development of round window opening tools (7). Different studies have evaluated in an artificial cochlea model insertional factors that could influence residual hearing, like force (8) and pressure, in terms of speed (9), moisturized insertion (4), manual tremor and automated insertion (10), and positioning of the cochlear array (11). The clinical importance of these findings has been underlined by various studies (12–14). Even the effects of several electrodes have been extensively evaluated (15–17).

Various techniques are performed at the round window during insertion in terms of the fluid situation. During cochleostomy as an approach to the cochlea, the usage of hyaluronic acid was popular in preventing blood and bone dust contamination of the cochlea. Recently, an underwater insertion (13) was introduced in which the hearing preservation effect was assumed to be based on the decreased pressure during a trans-fluid round-window opening (6) and a moisturizing effect (4).

The aim of the present study was to observe various fluid conditions in front of the round window during cochlear implant electrode insertion in a non-validated artificial model.

## Materials and Methods

### Model and Insertion Techniques

#### Pressure Sensor

We measured the intracochlear pressure (ICP) using a micro-fiber optical pressure sensor (FOP) (FISO, Canada). The tip of the pressure sensor is a hollow glass tube sealed on one end by a thin plastic film diaphragm coated with a reflective surface of evaporated gold. The optical fiber is located in the glass tube with a short distance (50–100 μm) to the diaphragm tip. The optical fiber is attached to a LED light source and a photodiode sensor. Light from the LED source reaches the sensor tip of the optical fiber, fans out as it exits the fiber, and is reflected by the gold-covered flexible diaphragm. The photodiode senses the reflected light, and small pressure-induced distance displacements of the diaphragm modulate the reflected light's intensity. The sensor is connected to a module that is linked to a computer. We used evolution software to record the ICP (FISO, Quebec, Canada). The time sensitivity of the sensor was 300 measurements per second.

#### Model

**Figure 1:**
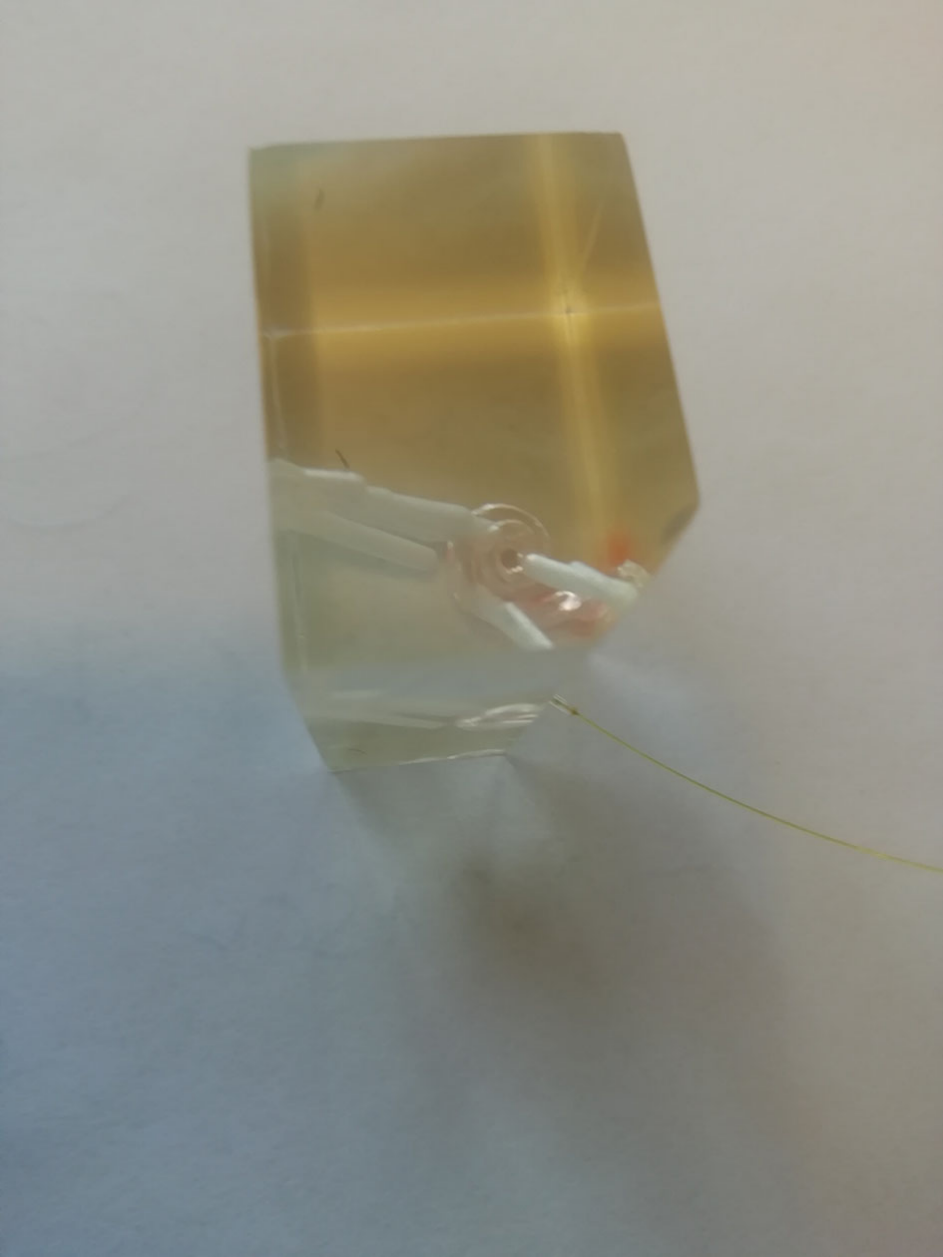
Insertional 3D-printed model with pressure sensor channels and a sensor in the basal turn of the scala tympani.

The model was a 3D-printed polyethylene artificial model of the scala tympani with a middle ear space. The model contains no basilar membrane influencing the cochlear hydrodynamics. We positioned the sensor into a hole burred into the tip of the apical cochlea. Figure 1 shows the 3D model with multiple burred channels into the cochlea and an inserted sensor. The sensor was sealed against the model to exclude passing out fluid. The used fluid inside the artificial cochlea was water.

#### Procedure

The electrode (Cochlear Slim Straight) was manually inserted in 30 s. Each experiment was performed five times. The control over the insertional time was given by a time assistance. During the insertions in the model, a second person measured in parallel the pressure changes caught by the sensor/controller/laptop unit.

We performed various series to observe different insertional aspects with the experimental setup. The experimental setup focused on the situation in front of the round window and the middle ear, because this is known to be important for the occurrence of pressure intracochlearly during the insertional procedure (17).

#### Experimental Setup

1. Hyaluronic acid:
  The artificial cochlea is filled with fluid. Viscous hyaluronic acid is placed in front of the round window. The insertion of the electrode is performed through the hyaluronic acid.
2. Wet electrode:
  The artificial cochlea is filled with fluid. The middle ear is completely dry. Only the electrode itself is moisturized with fluid, and the insertion is performed directly in the scala tympani.
3. Underwater:
  The artificial cochlea is filled with fluid. The middle ear is completely filled with fluid. The insertion of the electrode is performed through the fluid of the middle ear.
4. Indirect wet:
  The artificial cochlea is filled with fluid. The middle ear walls are wet. The electrode itself is moisturized, and the insertion is performed through a puddle of fluid close to the round window.

#### Analysis

We calculated the differences among the fluid condition-related pressure changes and statistically analyzed them using a one-way ANOVA and the Tukey *post-hoc* test (SPSS 24.00).

The institutional review board approved this study (Klibi-HNO-2020-001).

## Results

**Figure 2:**
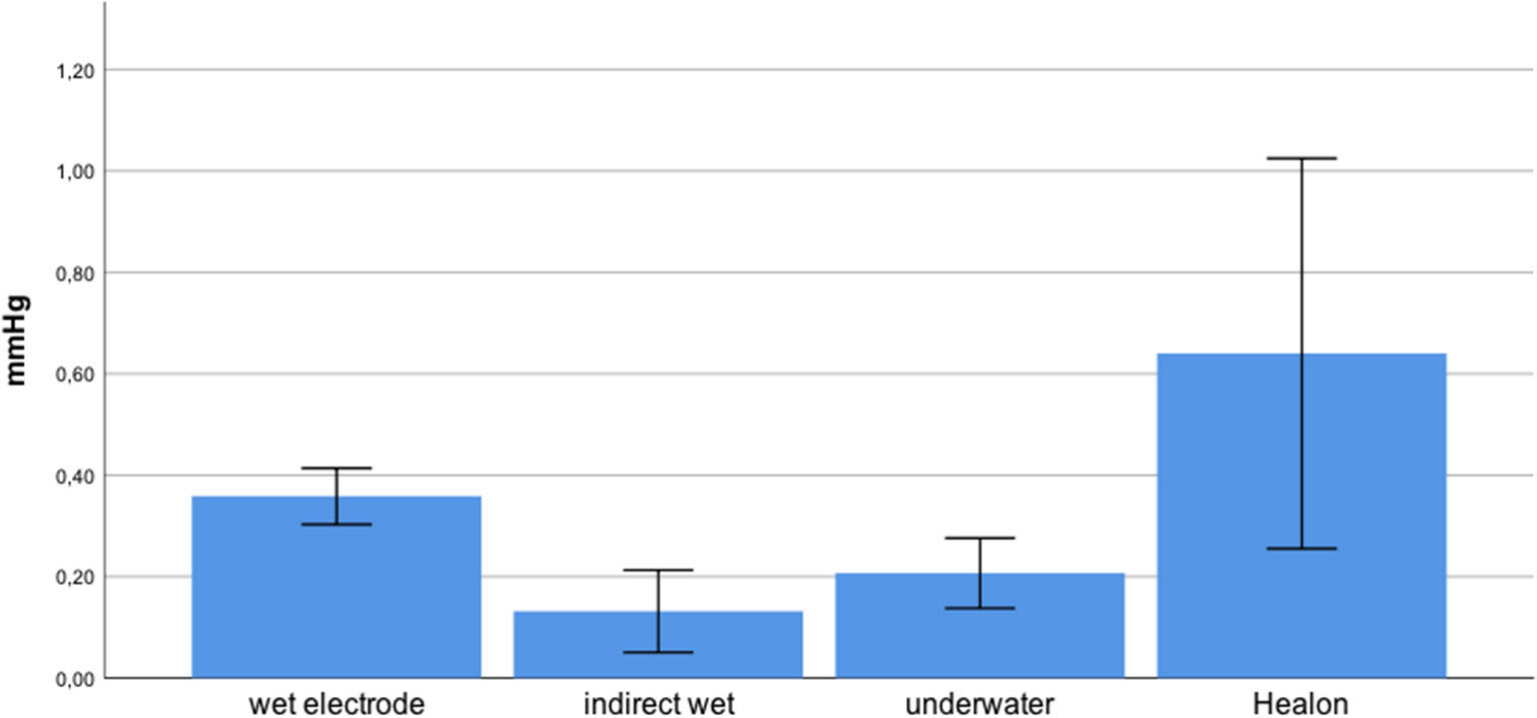
Mean pressure of various insertional modes with SD.

We presented data as mean ± standard deviation. The mean maximum intracochlear fluid pressure (ICFP) decreased from hyaluronic acid (0.64 ± 0.31) to wet electrode (0.36 ± 0.05), underwater (0.21 ± 0.06), and indirect wet (0.13 ± 0.07), in that order (Figure 2).

The differences between some of these techniques were statistically significant. We distributed the data for each group, as assessed by a Shapiro–Wilk test (*p* < 0.05). The homogeneity of variance was violated, as assessed by Levene's test of homogeneity of variance (*p* = 0.003).

**Figure 3:**
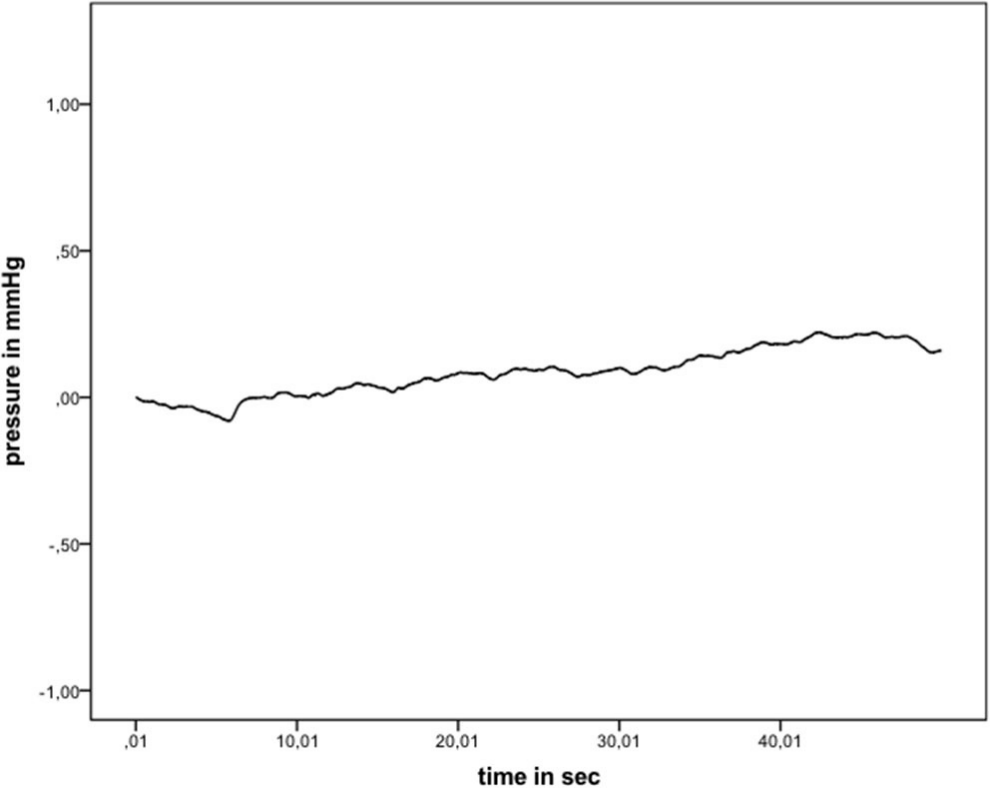
Exemplary pressure measurement (indirect wet).

Games–Howell *post-hoc* analysis revealed that the difference from hyaluronic acid to indirect wet was statistically significant (*p* = 0.02). This was also true from hyaluronic acid to underwater (*p* = 0.034), wet electrode to indirect wet (*p* < 0.001), and wet electrode to underwater (*p* = 0.001). Results of each performed experiment are presented in Table 1. Figure 3 shows an exemplary measurement.

**Table 1:** Results of all performed experiments of the four insertional conditions in mmHg.

	**Exp. 1**	**Exp. 2**	**Exp. 3**	**Exp. 4**	**Exp. 5**	**SD**
Wet electrode	0.36	0.39	0.33	0.41	0.3	±0.04
Indirect wet	0.05	0.22	0.16	0.14	0.09	±0.07
Underwater	0.18	0.18	0.28	0.25	0.15	±0.05
Healon	1.02	0.43	0.25	0.86	0.64	±0.31

## Discussion

Preservation of residual hearing is one of the central aims of modern cochlear implantation. Various experimental and clinical studies have underlined the role of ICP changes in this relationship. Pre-insertional, intra-insertional, and post-insertional factors have been shown experimentally to influence the ICP. Pre-insertional factors, such as the size of the round window opening (4), opening technique (5), and the fluid state of opening (6) have been shown to affect ICP. The intra-insertional automated procedure and decrease in tremors by supported insertion (10) have been shown to affect ICP, as well as the speed of insertion (9). Additionally, the choice of electrode array in terms of tip size and volume of electrode (15–17), as well as the depth of insertion in terms of fast pressure changes (18) and static pressure changes, has been described (19). Even the effect of specific insertional techniques, such as the electrode pullback (20) has been observed. Post-insertional evaluation of the effect of sealing techniques (21) and post-insertional cable movements (11) on ICP has been performed.

Clinically, the pre-insertional effects, such as the size of the round window opening (22) and fluid state in moisturization of the electrode, have been evaluated and have been found to significantly affect hearing preservation (14). The insertional speed, as an intra-insertional factor, has been shown to affect the rate of hearing preservation (12).

All these clinical results underline the importance of pressure transients on the preservation of residual hearing. The value besides other factors like insertional force and insertional angle needs to be further evaluated.

Recently, a so-called underwater technique (13, 23) was shown to have a positive effect on the rate of residual hearing. In this technique, the middle ear is filled with Ringer's solution, and the insertion of the electrode is performed through the fluid. To prevent blood or bone dust passing into the cochlea, hyaluronic acid is used in some clinics to block the cochlea before inserting the electrode.

We looked experimentally at this technique and observed a significant effect on ICP of the underwater technique in comparison to a purely moisturized electrode. Even in comparison to a hyaluronic-covered round window, we observed a significantly positive effect. It can be assumed that the viscous hyaluronic agent prevents the intracochlear fluid from passing out of the scala during insertion of the electrode. This might be the reason for the non-significant ICP value difference between the underwater mode and the indirect wet mode.

Based on this finding, indirect wet insertion showed the lowest ICP and can be recommended. Comparative clinical studies should be performed to clinically verify this experimental finding. Future investigation and validation of the model are advocated before implementation and translation to human cochlear implantation.

Limitations of the experimental model persist in terms of its ability to simulate natural pressure equilibration pathways like the cochlea aqueduct and the round window. Additional material differences between bone and model persist in terms of its adhesion abilities.

## Conclusion

The fluid state in front of the round window influences the ICP value during cochlear implant electrode insertion. Indirect insertion of a moisturized electrode through a wet middle ear experimentally generated the lowest pressure values. Hyaluronic acid in front of the round window leads to higher ICP during insertion in a non-validated artificial model.

## Data Availability Statement

The raw data supporting the conclusions of this article will be made available by the authors, without undue reservation.

## Author Contributions

CR: writing. HS: co-writing. IT: writing and experiments. All authors contributed to the article and approved the submitted version.

## Conflict of Interest

The authors declare that the research was conducted in the absence of any commercial or financial relationships that could be construed as a potential conflict of interest.
